# A case of giant Ewing's sarcoma (EES)/primitive neuroectodermal tumor (PNET) of the cervicothoracic junction in children with incomplete paralysis of both lower limbs: Case report and literature review

**DOI:** 10.3389/fsurg.2022.1066304

**Published:** 2023-01-06

**Authors:** Gong-Heng Zhang, Jun-Ming Lin, Zhen-Yu He, Xiao-Jun Yuan, Guang Li, Xin-Rong Gan, Wen-Hua Xu, Sheng-Sheng Cao

**Affiliations:** Department of Orthopaedic Surgery, Yichun People's Hospital, Yichun, China

**Keywords:** extraosseous Ewing's sarcoma/primitive neuroectodermal tumor, children, cervicothoracic junction, case report, paralysis

## Abstract

**Background:**

Extraosseous Ewing's sarcoma/primary neuroectodermal tumor (EES/PNET) is a rare, malignant, small round blue cell tumor, which usually involves the larynx, kidneys, and esophagus. The most common metastatic sites are lung and bone. The incidence of epidural EES/PNET was 0.9%, and a detailed search of the PubMed literature found only 7 case reports of epidural ESS/PNET at the cervicothoracic junction in children.

**Case description:**

We report a case of epidural ESS/PNET at the cervicothoracic junction in a child with chest and back pain as the first symptom, which worsened after half a year and developed incomplete paralysis of both lower extremities and urinary incontinence. She underwent emergency surgery, chemotherapy and radiotherapy, and died of lung metastases 8 months after surgery.

**Conclusion:**

Primary epidural tumors are mostly benign, such as spinal meningiomas and neuromas. Contrary to what has been previously thought, we report a case of malignant epidural EES/PNET at the cervicothoracic junction without bone destruction; The rarity of epidural EES/PNET at the cervicothoracic junction in children has led to a lack of data, particularly on prognostic factors and recurrence patterns. Due to the difficulty of early diagnosis and high mortality, spine surgeons must explore and increase their awareness of this disease.

## Introduction

Ewing sarcoma (ES) and primitive neuroectodermal tumors (PNET) are collectively referred to as ES/PNET because they share the same genetic and histological features (1). ES/PNET are highly malignant small round cell tumors with multidirectional differentiation potential and can be classified as intraosseous and extraosseous depending on their origin in bone or soft tissue (2). Extraosseous Ewing's sarcoma (EES)/PNET is an extremely rare primary malignant tumor with rapid progression and poor prognosis (3). EES/PNET has been reported mainly in the chest wall, lower extremities and paravertebral regions, followed by the upper extremities, hip and pelvis, and rarely in the epidural space, especially at the cervicothoracic junction (4, 5).

To date, the involvement of the epidural space in the cervicothoracic junction is rarely described in the literature, and there are only a few cases reported worldwide. The rarity of epidural EES/PNET at the cervicothoracic junction in children has led to a lack of data, particularly on prognostic factors and recurrence patterns. We report a rare case of cervicothoracic junction EES/PNET in a 12-year-old boy. Surgical resection was performed with postoperative radiotherapy and chemotherapy. The entire clinical diagnosis and treatment process is reported, and our case is compared with other previously reported cases.

## Case report

**Figure 1:**
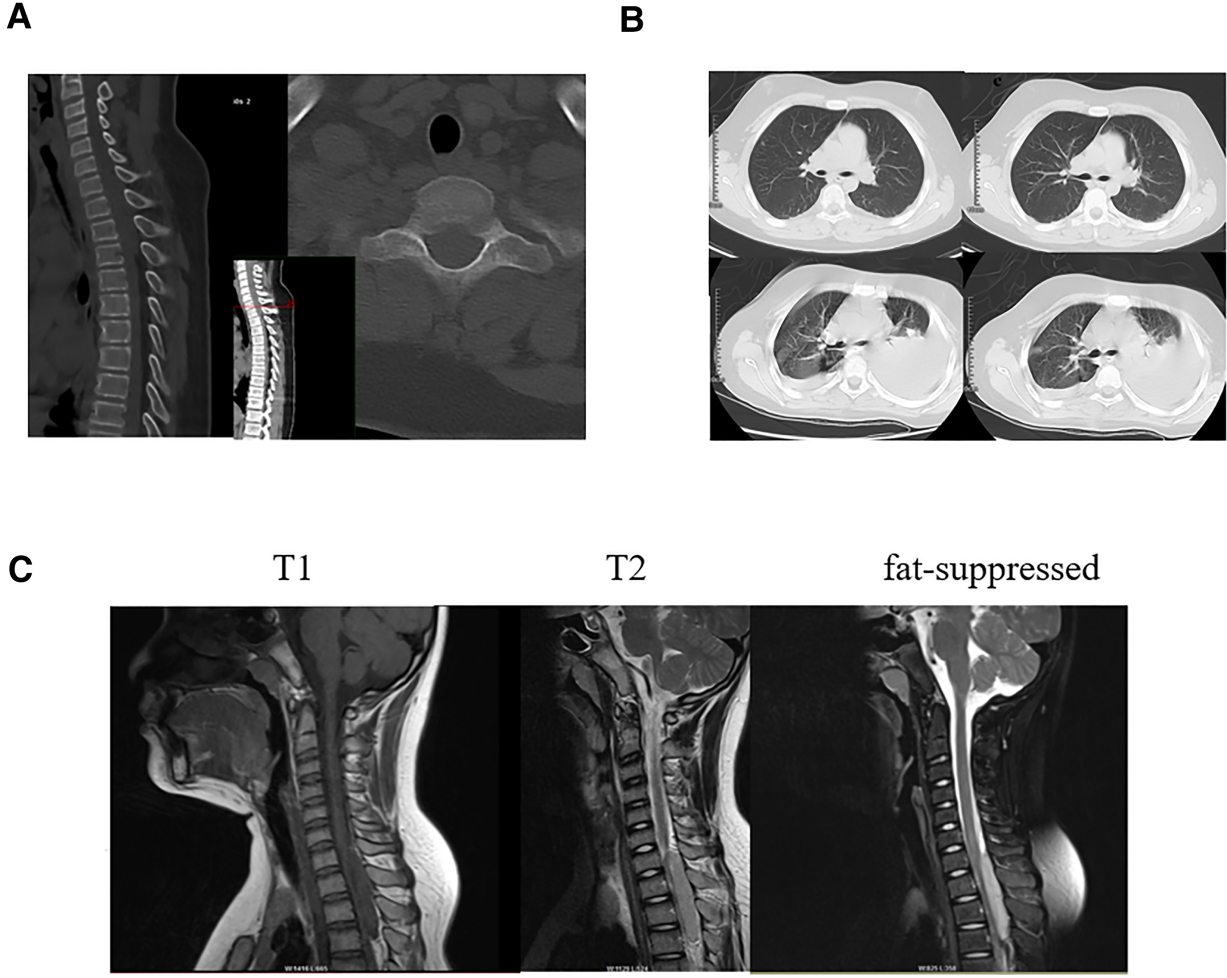
(**A**) Preoperative transverse and sagittal CT showed no bone destruction in the cervicothoracic junction. (**B**) Preoperative lung CT showed no obvious signs of tumor metastasis,Six months after the operation, lung CT showed multiple nodules in both lungs, and metastasis was considered. (**C**) T1-weighted, T2-weighted, and fat-suppressed sagittal MRI showed a long tubular mass in the spinal canal at the cervicothoracic junction, with a size of about 8 mm × 15 mm × 63 mm, and no abnormal signal vertebral body and appendage morphology.

A 12-year-old boy who appeared to be healthy developed chest and back pain. His symptoms improved after treatment with nonsteroidal anti-inflammatory drugs (NSAIDs) at a local hospital. however, his condition, gradually worsened within 6 months. One day ago, he was transferred to our hospital for sudden incomplete paralysis of both lower limbs, and urinary incontinence. He had no history of any trauma or cerebrovascular accident. On examination, he was conscious with normal higher mental functions. Motor system examination showed that the strength of the upper limbs was MRC grade 5/5, and the strength of the lower limbs was only MRC grade 1/5. The tone of the muscles of the upper limbs was normal, but the tone of the muscles of the lower limbs was decreased. The skin sensation below the T3 plane was decreased. The deep tendon reflexes of the patella and Achilles tendon were impaired, and the Babinski sign was positive on both sides. The JOA score was 8 points. Preoperative transverse and sagittal CT showed no bone destruction in the cervicothoracic junction, and CT of the lungs showed no significant abnormalities. MRI showed a long soft tissue mass at the cervicothoracic junction (C7-T3) with the spinal cord compressed and shifted to the right. It was isointense on T1W and hyperintense on T2W images. MRI showed that the tumor in the cervicothoracic spinal canal was approximately 8 mm × 15 mm × 63 mm (Figure 1).

**Figure 2:**
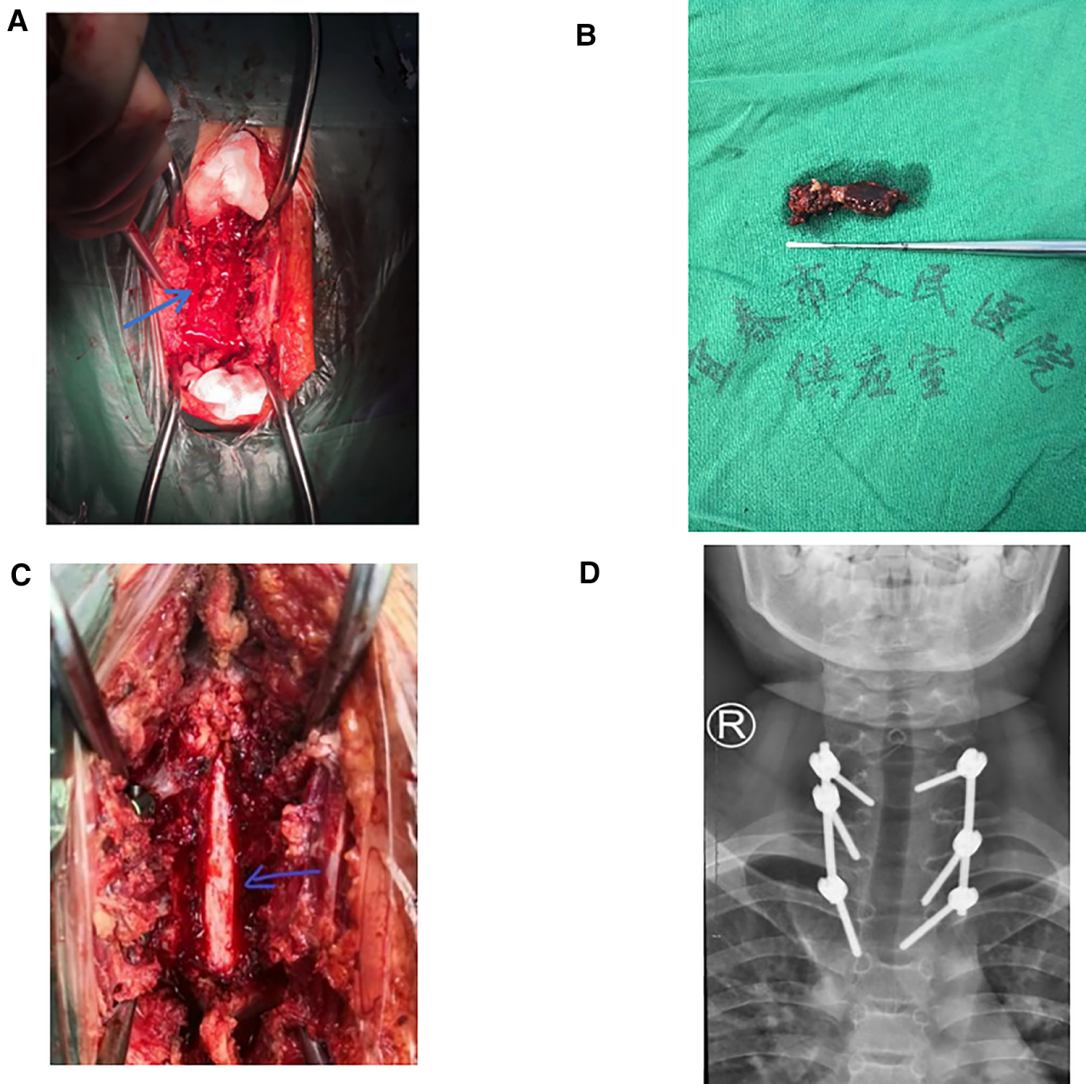
(**A**) Severe dural sac compression before surgery, (**B**) intraoperative excision of the tumor, about 5 cm long, (**C**) the dural sac was entire and pulsating after tumor resection, (**D**) postoperative radiographs showed accurate pedicle screw placement.

**Figure 3:**
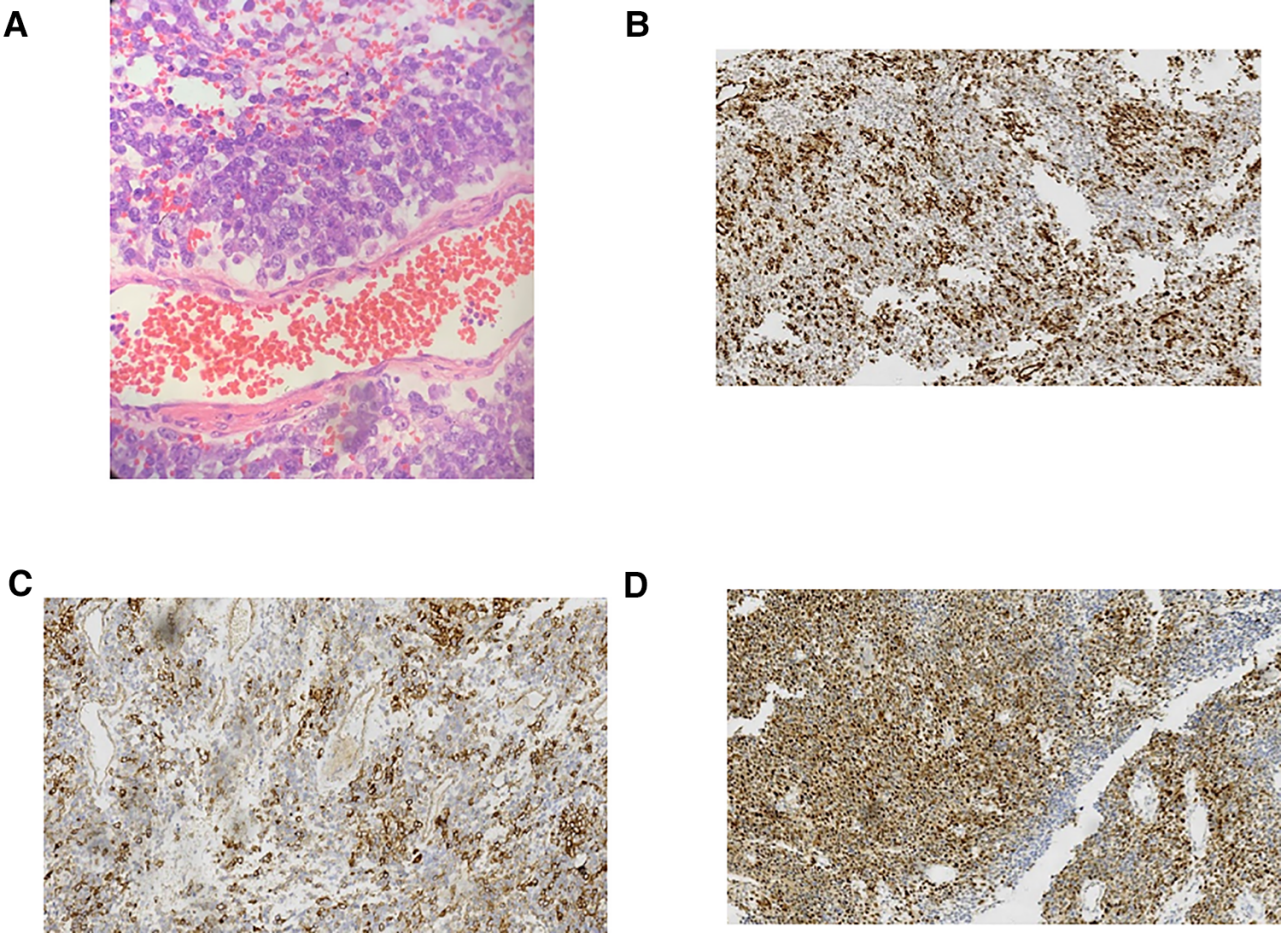
(**A**) Pathological examination microscope showed small round blue cells (HE staining, ×200). (**B–D**) Immunohistochemical staining showed that vimentin (**B**), CD99 (**C**) and Cyclin D1 (**D**) were strongly positive in small round blue cells (×100).

Due to the sudden onset of symptoms of bilateral lower limb incomplete paralysis, preoperative biopsy and chemotherapy were not performed. After ruling out contraindications to surgery, an emergency C7-T3 laminectomy was performed. Pedicle screws were placed on both sides of C7 and T3, the right side of T1 and the left side of T2 to fix the unstable cervical spine.C7 and T3, the right side of T1 and the left side of T2 to fix the unstable cervical spine (Figure 2). Intraoperatively, a flesh-like tumor with soft texture and rich blood supply was found in the epidural space. It filled the entire vertebral cavity of C7-T3 and extruded the dura mater to the ventral side. The tumor was completely separated out and excised along the envelope under microscopic manipulation, the dura mater was intact and pulsatile, and the lesion was biopsied (Figure 2). Immunohistochemistry showed positivity for Ki67, strong positivity for Vimentin, Cyclin D1, and CD99, and negativity for CD56, EMA, CKpan, Desmin, LCA, CD34, NKX2.2 and Syn. The histopathological report showed malignant small round blue tumors, which is consistent with Ewing's sarcoma/PNET (Figure 3). The patient's family decided not to perform next-generation sequencing (NGS) due to the high cost of the test.

Postoperative motor system examination showed that the strength of the right lower limbs was MRC grade 3/5, and the strength of the left lower limbs was MRC grade 1/5. The JOA score was11 points. After discussion with the oncologist two weeks after the surgery, it was decided to give him chemoradiotherapy. First-line palliative chemotherapy with vincristine (VCR), doxorubicin (DOX), and cyclophosphamide (CPA) was initiated in June 2019. We planned 1.5 mg/m^2^ of VCR per week, 30 mg/m^2^ of DOX per day and 1.2 mg/m^2^ of CPA per day. After two cycles we implemented ifosfamide and etoposide as alternative treatment. We planned 1,800 mg/m^2^ of ifosfamide per day and 100 mg/m^2^ of etoposide per day for five days. Pulmonary metastases occurred after 6 months of follow-up with no local recurrence (Figure 1). Motor system examination showed MRC grade 3/5 strength in the right lower extremity and MRC grade 2/5 strength in the left lower extremity. The treatment group discussed the treatment plan with the patient's family, the patient's family decided not to pursue additional treatment, and he died 8 months after surgery. The course of the disease diagnosis and treatment is shown in the timeline.


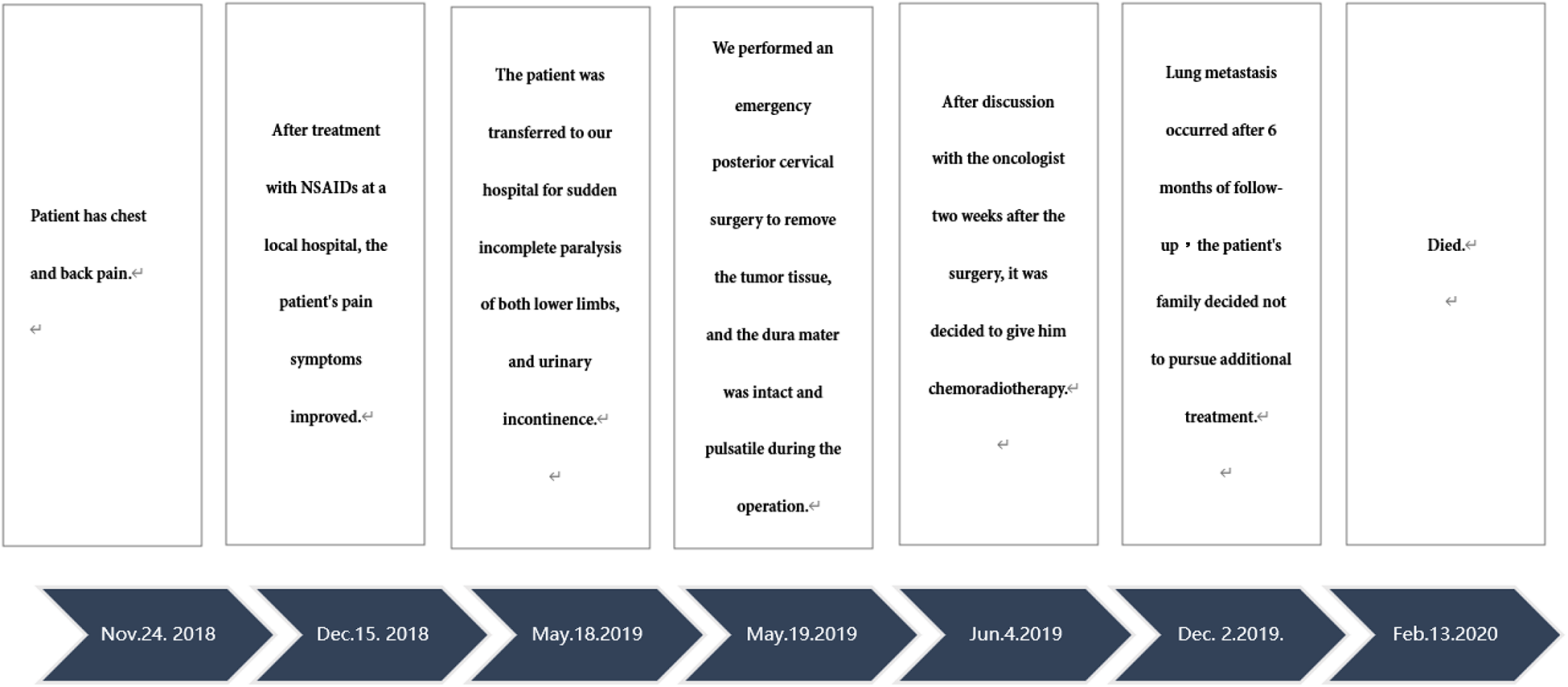
Timeline: NSAIDs: Nonsteroidal anti-inflammatory drugs

## Discussion

EES is a rare, malignant, small round blue cell tumor of undifferentiated mesenchymal origin, originating from the neural crest of the central and sympathetic nervous systems. It was first reported by Tefft et al. in 1969. Both PNET and Ewing's sarcoma (ES) have the same small round blue cells and similar molecular relationships. In most literature reports, researchers describe these entities together as EES/PNET (6). EES/PNET has been reported in various sites, such as the larynx, kidneys, and esophagus. The presence of EES/PNET in the extradural area is very rare, with an incidence of 0.9% (7). Pediatric spinal epidural EES/PNET is a very rare tumor. There have been less than 50 cases reported thus far. The most common site was the lumbar spine. Cervical and thoracic spines are rare sites, and males are most commonly affected (8). A detailed search of the literature in PubMed revealed only 8 cases of epidural ESS/PNET in the cervicothoracic junction in children, and the ratio of males to females was 1.67 : 1 as previously reported (Table 1).

**Table 1:** Case data of ESS/PNET at the cervicothoracic junction in children.

Time	Age	Gender	Location	Treatment	Follow-up (months)	Ending
2001 (9)	13	Female	C7-T1	Chemotherapy, radiotherapy		
2002 (10)	5	Male	C7-D1	Surgery, radiotherapy	8	No recurrence
2003 (11)	10	Male	C6-T3	Surgery, chemotherapy, radiotherapy	22	Death
2007 (12)	7	Male	C5-T1	Surgery, chemotherapy, radiotherapy	90	No recurrence
2011 (13)	7	Male	C6-T2	Surgery, chemotherapy, radiotherapy	6	No recurrence
2016 (14)	12	Female	C5-D1	Surgery, radiotherapy	6	Death
	11	Female	C6-C7	Surgery, chemotherapy, radiotherapy	25	Death
2020 (this research)	12	Male	C7-T3	Surgery, radiotherapy	8	Death

In general, the intradural and extramedullary EES/PNET will not extend into the adjacent vertebrae and tends to infiltrate the paravertebral muscle tissue or neural foramina (6). This can potentially cause nerve damage, resulting in loss of sensory or motor function of the arms and legs (paraplegia or quadriplegia). Pain and muscle weakness corresponding to the tumor location are the most common clinical features of EES/PNET (15, 16). Our patient had only chest pain before incomplete paralysis of both lower extremities, and the failure to receive timely and accurate treatment was considered to be a factor in the poor prognosis of the patient.

The differential diagnosis of EES/PNETs in children includes Hodgkin’s disease, neuroblastoma, rhabdomyosarcoma, and Wilms’ tumor (17). Early diagnosis of EES/PNETs was mainly based on MRI, but it was considered difficult to distinguish EES/PNET from other tumors originating in the spinal cord based on depending upon the MRI signal intensity alone (13, 18). On MRI, EES/PNET was usually isointense to the muscles on T1W and hyperintense on T2W images, with enhancement on postcontrast scans. The histopathological biopsy features of EES/PNET consist of uniform small round blue cells, but they often need to be distinguished from other small round cell malignancies, which can easily be misdiagnosed as lymphoma (19). These small round blue cells often show immunohistochemical positivity for CD99, vimentin, and cyclin D1, as in our case report.

At present, neoadjuvant radiotherapy and chemotherapy combined with *en bloc* resection is the gold standard for treating limb Ewing's sarcoma (20). However, when the intradural and extramedullary EES/ PNET compress the spinal cord, radiotherapy and chemotherapy may cause damage to the spinal cord nerves, and immediate decompression surgery may be necessary (21). In our case, the patient needed emergency decompression due to sudden numbness and weakness of both lower limbs. Then C7-T3 laminectomy was performed as an emergency operation. Moreover, *en bloc* resection is the standard procedure for EES/PNET, but in practice, most epidural EES/PNET cases are partially excised to preserve the nerve and await a definitive pathological diagnosis. The most common metastatic sites of EES/PNET are the lung and bone. In our case, after the complete removal of tumor tissue, the dura was intact and pulsatile. Postoperative radiotherapy and chemotherapy. Lung metastasis occurred after 6 months of follow-up, with no local recurrence, and died 8 months after surgery.

EES/PNET in the epidural space of the cervicothoracic junction is a malignant tumor with rapid progression and poor prognosis. We found, through the literature review, that a total of 7 children (including the case in this study) were followed up, and the case fatality rate was 57% (4/7) (Table 1). Factors involved in prognosis include tumor location, tumor volume, tumor resection rate, treatment response, time of onset, and presence of metastasis. The cause of death may be associated with an onset of symptoms 6 months prior to diagnosis and, a large tumor that, is located in the epidural space of the cervicothoracic junction. Incomplete surgical tumor resection may also be a reason for the high recurrence rate and low survival rate of patients (5, 22). In our case, since PET-CT was not performed, the patient may have had bone metastases in other parts of the body at the first admission. This may be the main reason for the occurrence of late lung metastases. Another reason may be the increased risk of secondary cancers, mainly treatment-induced acute myeloid leukemia (t-AML) or radiation-induced sarcomas, and breast and thyroid cancers, in pediatric EES/PNET patients after treatment (23). The lack of NGS technology to further clarify the pathological classification of Ewing's sarcoma may also contribute to the poor prognosis of patients.

## Conclusion

Primary epidural tumors are mostly benign, such as spinal meningiomas and neuromas. Contrary to what has been previously thought, we report a case of malignant epidural EES/PNET at the cervicothoracic junction without bone destruction. Preoperative PET-CT scanning is important to determine whether the tumor has metastasized to other organs and to assess the patient's survival prognosis.

## Data Availability

The original contributions presented in the study are included in the article/Supplementary Material, further inquiries can be directed to the corresponding author.
